# Validity of DXA body volume equations in a four-compartment model for adults with varying body mass index and waist circumference classifications

**DOI:** 10.1371/journal.pone.0206866

**Published:** 2018-11-05

**Authors:** Cherilyn N. McLester, Brett S. Nickerson, Brian M. Kliszczewicz, Courtenay S. Hicks, Cassie M. Williamson, Emily E. Bechke, John R. McLester

**Affiliations:** 1 Department of Exercise Science and Sport Management, Kennesaw State University, Kennesaw, Georgia, United States of America; 2 College of Nursing and Health Sciences, Texas A&M International University, Laredo, Texas, United States of America; Liverpool John Moores University, UNITED KINGDOM

## Abstract

The purpose of this investigation was to determine the validity of 4-compartment (4C) model body fat percent (BF%) estimates when using dual energy x-ray absorptiometry (DXA) derived body volume (BV) equations (4C-DXA1 and 4C-DXA2) in adults with varying body mass index (BMI) and waist circumference (WC) classifications. Each model was compared to a criterion 4C model with air-displacement plethysmography (ADP) generated BV (4C-ADP). Participants were categorized as normal weight (n = 40; NW = BMI<25.0kg/m^2^); overweight (n = 40; OW_BMI_ = BMI≥25.0 kg/m^2^); and overweight with at-risk WC (n = 35; OW_BMI+WC_ = BMI≥25.0 kg/m^2^ and WC≥88.0cm for women and 102.0cm for men). 4C-DXA1 produced lower BF% than that derived using the 4C-ADP in NW (CE = -3.0%; p<0.001) while 4C-DXA2 was significantly higher (CE = 4.8%; p<0.001). The SEE and 95% limits of agreement (LOA) were lower for 4C-DXA2 (1.24% and ±2.5%, respectively) than 4C-DXA1 (2.59% and ±5.0%, respectively) and proportional bias was present for both (p<0.05). 4C-DXA1 BF% was not significant in OW_BMI_ (CE = -0.5%; p = 0.112) whereas 4C-DXA2 was higher (CE = 4.5%; p<0.001). The SEE and 95% LOA were lower for 4C-DXA2 (1.20% and ±2.9%, respectively) than 4C-DXA1 (1.92% and ±3.9%, respectively) in OW_BMI_. Proportional bias was present for 4C-DXA1 (p = 0.007), but not 4C-DXA2 (p = 0.832). 4C-DXA1 and 4C-DXA2 produced significantly higher BF% in OW_BMI+WC_ (CE = 2.2 and 2.3%, respectively; both p<0.001). The SEE and 95% LOA remained lower for 4C-DXA2 (1.15% and ±2.5%, respectively) than 4C-DXA1 (1.84% and ±3.8%, respectively). There was proportional bias for 4C-DXA2 (p = 0.020), but not 4C-DXA1 (p = 0.183) in OW_BMI+WC_. Only one prediction model (i.e., 4C-DXA1 in OW_BMI+WC_) revealed valid estimates of BF%. Practitioners are encouraged to use criteria for both BMI and WC when utilizing DXA-derived BV in 4C-models for normal and overweight populations.

## Introduction

Body composition is a fundamental measure for health, fitness, and risk assessment utilized by practitioners and fitness professionals alike. Information provided by various body composition techniques has proven beneficial in the stratification of risk for cardiovascular and metabolic diseases [1, 2]. Further, body composition among obese individuals varies widely with greater health risk associated with those carrying more adipose tissue and less lean tissue [3]. In more applied settings, accurate body composition provides information used to assess the effectiveness of clinical interventions and overall behavior modifications. The significance of accurate measures of body composition has led to improvements in the techniques used to quantify and interpret these measurements. From early assessments such as skinfolds and single-frequency bioelectrical impedance analysis (BIA) along with the simpler measures of body mass index (BMI) and waist circumference (WC), several advanced techniques are now available. These techniques include body volume (BV) measures via air displacement plethysmography (ADP) or under water weighing (UWW), bone mineral content (BMC) via dual-energy x-ray absorptiometry (DXA), or total body water (TBW) analysis via multi-frequency BIA.

Each technique provides a unique aspect of body composition and the combination of these metrics allows the practitioner to conduct a more comprehensive assessment. Specifically, the use of a four-compartment (4C) model has become an accepted method and is commonly used as a criterion in body composition research [4–7]. The use of a 4C model requires the measurements of BV (ADP or UWW), BMC (DXA), and TBW (BIA) [8]. Despite the improved precision of a 4C model derived with traditional methods (e.g., ADP for BV, DXA for BMC, and BIA for TBW), research continually seeks to identify the validity of modified 4C models when traditional methods are not fully accessible [9, 10].

Recently, researchers have developed two separate DXA-derived BV equations in order to replace the need for traditional displacement techniques from UWW or ADP [11, 12]. Empirical evidence of the newly derived DXA BV equations is limited. For instance, Nickerson et al. [9] used a criterion UWW for BV in a 4C model and reported that the DXA-derived equation from Smith-Ryan et al. [11] should not be used in a 4C model for physically active adults. In contrast, Blue et al. [13] revealed the Smith-Ryan et al. [11] equation to be valid in a 4C model when compared to using ADP derived BV in adults classified as overweight/obese (i.e., BMI ≥ 25 kg/m^2^).

The variance among the previous findings utilizing the Smith-Ryan et al. [11] DXA BV equation is likely due to differences in participant characteristics. The Smith-Ryan et al. [11] equation was developed with subjects with an average BMI classified as obese (31.4 ± 5.5 kg/m^2^), which is similar to those in the Blue et al. [13] population. The noticeable differences in both study populations raise the question as to whether the validity of DXA BV equations in a 4C model is influenced by participants with varying BMI levels. It is fairly common for someone that is physically active to have recommended adiposity levels and high quantities of fat-free mass, but be categorized as overweight/obese via BMI. As a result, the classification of normal weight or overweight might be more appropriate when using the criteria for both BMI and WC. Previous research from Nickerson et al. [9] and Blue et al. [13] did not include the WC of participants. Furthermore, the validity of the Wilson et al. [12] DXA BV equation in a 4C model has yet to be examined. Therefore, the purpose of the current study was to determine the validity of 4C model BF% estimates when using DXA-derived BV equations in adults with varying BMI and WC classifications.

## Materials and methods

### Participants

Kennesaw State University Institutional Review Board approved this study and written consent was obtained by each participant prior to any data collection. A total of 115 individuals volunteered for this study. In order to qualify, participants needed to be between the ages of 18–65 years. Exclusion criteria included pregnant women, along with those missing limbs or with pacemaker implants. Subjects were split into three different groups; 1) normal weight (n = 40; NW = BMI < 25.0 kg/m^2^ and WC < 88.0 and 102.0 cm for women and men, respectively); 2) overweight (n = 40; OW_BMI_ = BMI ≥ 25.0 kg/m^2^); 3) overweight (n = 35; OW_BMI+WC_ = BMI ≥ 25.0 kg/m^2^ and WC ≥ 88.0 and 102.0 cm for women and men, respectively). Participant characteristics are displayed in Table 1.

**Table 1:** Subject characteristics (mean ± standard deviation).

Males	Normal Weight (N = 20)	Overweight_BMI_ (N = 20)	Overweight_BMI+WC_ (N = 14)
Age (years)	23.2 ± 2.9	26.5 ± 9.1	34.6 ± 10.2
Height (cm)	178.4 ± 5.8	178.6 ± 9.7	179.1 ± 8.5
Body Mass (kg)	74.1 ± 6.3	89.4 ± 11.9	120.4 ± 20.4
Body Mass Index (kg/m^2^)	23.1 ± 1.5	28.1 ± 2.8	37.5 ± 4.8
Waist Circumference (cm)	76.8 ± 4.2	86.3 ± 6.6	112.0 ± 10.8
4C-ADP FFM (kg)	65.9 ± 6.1	73.2 ± 8.2	78.1 ± 12.0
4C-ADP FM (kg)	8.2 ± 3.2	16.2 ± 8.2	42.3 ± 15.4
4C-ADP BF%	11.0 ± 4.1	17.6 ± 7.3	34.5 ± 8.5
Females	Normal Weight (N = 20)	Overweight_BMI_ (N = 20)	Overweight_BMI+WC_ (N = 21)
Age (years)	26.4 ± 8.2	22.0 ± 2.8	36.9 ± 13.2
Height (cm)	163.6 ± 6.5	162.6 ± 7.2	165.4 ± 5.3
Body Mass (kg)	57.2 ± 7.8	71.1 ± 6.6	104.7 ± 20.7
Body Mass Index (kg/m^2^)	24.1 ± 1.7	26.9 ± 1.6	38.4 ± 8.0
Waist Circumference (cm)	67.2 ± 4.7	77.0 ± 5.1	103.8 ± 14.7
4C-ADP FFM (kg)	45.2 ± 6.2	47.1 ± 6.3	57.3 ± 7.9
4C-ADP FM (kg)	12.0 ± 3.6	24.0 ± 4.3	47.4 ± 15.3
4C-ADP BF%	20.8 ± 5.3	33.9 ± 5.7	44.5 ± 6.2

### Procedures

A single visit lasting approximately 45–60 minutes was required for each participant and all assessments were completed within the university’s exercise physiology laboratory. In order to ensure standardized conditions, each participant was instructed to abstain from exercising and consuming alcohol for 24 hours and to abstain from eating or drinking, with the exception of water, for four hours prior to testing. Lastly, they were instructed to wear minimal, tight fitting, lycra attire that was free from metal. In the case that a participant did not have the proper attire, tight fitting lycra shorts and sports bra were provided as needed. Upon arrival for testing, participants completed a written consent form and a demographic questionnaire that gathered information on sex and race.

Following the questionnaire participants were asked to void their bladder and were then instructed to remove extra clothing, shoes, metal, watches, wristbands, and jewelry. Height and weight were measured with a Tanita WB-3000 (Tanita, Arlington Heights, IL, USA) digital physician’s scale and BMI was calculated from height and weight measurements (kg/m^2^). Each participant then completed three different body composition assessments which included dual-energy X-ray absorptiometry with the GE Lunar iDXA (General Electric, Madison, WI, USA), air-displacement plethysmography with the BodPod (COSMED USA Inc, Concord, CA, USA), and bioelectrical impedance analysis (BIA) with the InBody720 (InBodyUSA, Cerritos, CA, USA). Finally, WC was obtained from each participant (cm).

#### Dual-energy X-ray absorptiometry (DXA)

The iDXA was used to calculate BMC for 4C-ADP and to estimate BV and BMC via 4C-DXA1 and 4C-DXA2. Each subjects’ BMC was converted to Mo, which is total body bone mineral with the following equation: (*M_o_* = *BMC* * 1.0436) [14]. Quality assurance was conducted daily per manufacturer’s specifications. Participants were tested per manufacturer specifications. Regions of interest were evaluated by the primary investigator and adjusted if needed. DXA derived BV was determined with the following equations from Smith-Ryan et al. [11] and Wilson et al.[12]:

$${DXA-BV}_{SMITH-RYAN}\left(L\right)=\left(\frac{FM}{0.84}\right)+\left(\frac{LM}{1.03}\right)+\left(\frac{BMC}{11.63}\right)-3.12\;L$$

$${DXA-BV}_{WILSON}(L)=(0.95*LM)+(1.14*FM)+(0.21*BMC)+0.01\;L$$

#### Air displacement plethysmography

The BodPod, which uses air displacement plethysmography, was used to estimate criterion BV (i.e., 4C-ADP). Prior to testing each day, the BodPod was calibrated according to manufacturer specifications. In addition to required attire, lycra swim-caps were provided and worn for testing. Body mass was measured using the manufacturer’s scale that interfaces with the BodPod. In order to assess body volume participants were instructed to sit in the BodPod chamber for 2 trials of roughly 50 seconds for each trial. A third trial was necessary if the first two trials did not agree within 150 ml of each other. Body volume obtained from the BodPod was used for 4C-ADP BV estimates.

#### Bioelectrical impedance analysis (BIA)

The InBody720 was used to calculate TBW for all 4C models. The BIA device utilizes a tetrapolar 8-point tactile electrode system and takes 30 impedance measurements with six frequencies (1 kHz, 5 kHz, 50 kHz, 250 kHz, 500 kHz, and 1000 kHz) over a test duration of approximately 60 seconds. Prior to testing, participants removed their socks and cleansed their hands and feet with an antibacterial tissue purchased from the manufacturer. Total body water obtained from the InBody720 was used in all 4C models.

#### Waist circumference

Waist circumference was obtained per American College of Sports Medicine guidelines [15]. A spring-loaded Gulick tape measure (Fabrication Enterprises, White Plains, NY, USA) was used for all measurements. Participants were asked to stand with feet together and arms at their sides. A horizontal measurement was taken at the narrowest portion of the torso above the umbilicus and below the xiphoid process. Two measurements were taken and a third was obtained if the first two were not within 5 mm of each other. The criteria for those at a greater health risk was ≥ 88.0 cm for women and ≥ 102.0 cm for men [15].

#### Four-compartment model calculations

The equations developed by Wang et al. [8] were used for the 4C model:

$$FM(kg)=(2.748*BV)-(0.699*TBW)+(1.129*M_{o})-(2.051*BM)$$

$$BF\%=\left(\frac{FM}{BM}\right)*100$$

The 4C model equation utilizing the DXA derived BV from the Smith-Ryan et al. [11] equation is designated 4C-DXA1, and the 4C model equation utilizing the DXA derived BV from the Wilson et al. [12] equation is designated 4C-DXA2.

### Statistical analysis

The validity of 4C-DXA1 and 4C-DXA2 in all three groups were based upon the evaluation of predicted values versus the criterion (4C-ADP) by calculating the constant error (CE), *r* value, standard error of estimate (SEE), total error (TE), and proportional bias [16]. The differences in mean BF% (i.e., CE) among the prediction models and the 4C-ADP were analyzed using dependent t-tests (SPSS version 24) with the Bonferroni-adjusted alpha level (p ≤ 0.025). The magnitude of the effect size (ES) was determined by Hopkins’ scale [17] as follows: 0–0.2 = trivial, 0.2–0.6 = small, 0.6–1.2 = moderate, 1.2–2.0 = large, >2.0 = very large. The following thresholds were used to describe the *r* values: 0 to 0.30 small, 0.31 to 0.49 moderate, 0.50 to 0.69 large, 0.70 to 0.89 very large, and 0.90 to 1.00 near perfect [17]. The method of Bland-Altman [18] was used to identify the 95% limits of agreement of the BF% for the prediction models (4C-DXA1 and 4C-DXA2) and 4C-ADP. Linear regression was utilized to evaluate proportional bias between 4C-ADP and the prediction models using the procedures described by Tinsley [19].

## Results

### Normal weight

The validity of 4C-DXA1 and 4C-DXA2 when compared to 4C-ADP are displayed in Table 2. The mean BF% for 4C-DXA1 was significantly lower than that derived using the 4C-ADP (CE = -3.0%; p < 0.001) whereas 4C-DXA2 was significantly higher (CE = 4.8%; p < 0.001). The correlation coefficients were near perfect for both models while the TE was higher for 4C-DXA2 (4.99%) than 4C-DXA1 (3.99%). The SEE and 95% limits of agreement were lower for 4C-DXA2 (1.24% and ±2.5%, respectively) than 4C-DXA1 (2.59% and ±5.0%, respectively). The regression coefficients were statistically significant and demonstrated a proportional bias for both prediction models (p < 0.05).

**Table 2:** Comparison of BF% values between the 4C prediction models and 4C-ADP.

								95% Limits of Agreement		Linear Regression	
Method	(Mean± SD)	p-value	Cohen’s d	r	SEE	TE	CE ± 1.96 SD	Upper	Lower	Coefficient	p-value
**Normal Weight (n = 40)**	—	—	—	—	—	—	—	—	—	—	—
4C-ADP	15.9 ± 6.8	—	—	—	—	—	—	—	—	—	—
4C-DXA1	13.0 ± 6.0	< 0.001	0.45	0.927	2.59	3.91	-3.0 ± 5.0	2.0	-8.0	-0.132	0.042
4C-DXA2	20.7 ± 6.2	< 0.001	0.74	0.984	1.24	4.99	4.8 ± 2.5	7.4	2.3	-0.097	0.002
**Overweight**_**BMI**_ **(n = 40)**	—	—	—	—	—	—	—	—	—	—	—
4C-ADP	25.8 ± 10.5	—	—	—	—	—	—	—	—	—	—
4C-DXA1	25.2 ± 9.6	0.112	0.17	0.983	1.92	2.05	-0.5 ± 3.9	3.4	-4.5	-0.085	0.007
4C-DXA2	29.7 ± 9.4	< 0.001	0.23	0.994	1.20	4.69	4.5 ± 2.9	7.4	1.5	-0.005	0.832
**Overweight**_**BMI+WC**_**(n = 35)**	—	—	—	—	—	—	—	—	—	—	—
4C-ADP	40.5 ± 8.7	—	—	—	—	—	—	—	—	—	—
4C-DXA1	42.7 ± 9.1	< 0.001	0.25	0.978	1.84	2.88	2.2 ± 3.8	6.0	-1.5	0.050	0.183
4C-DXA2	42.8 ± 8.1	< 0.001	0.27	0.991	1.15	2.60	2.3 ± 2.5	4.8	-0.2	-0.076	0.020

BF% = body fat percentage; SEE = standard error of estimate; CE = constant error; TE = total error; SD = standard deviation.

### Overweight_BMI_

The mean BF% for 4C-DXA1 was not statistically significant when compared to that derived using the 4C-ADP (CE = -0.5%; p = 0.112) whereas 4C-DXA2 was significantly higher (CE = 4.5%; p < 0.001). The correlation coefficients were near perfect for both models although the TE was higher for 4C-DXA2 (4.69%) than 4C-DXA1 (2.05%). The SEE and 95% limits of agreement were lower for 4C-DXA2 (1.20% and ±2.9%, respectively) when compared to 4C-DXA1 (1.92% and ±3.9%, respectively). The proportional bias for 4C-DXA2 was not statistically significant (p = 0.832). However, the proportional bias for 4C-DXA1 was statistically significant (p = 0.007).

### Overweight_BMI+WC_

The mean BF% for 4C-DXA1 and 4C-DXA2 were significantly higher than that derived using the 4C-ADP (CE = 2.2 and 2.3%, respectively; both p < 0.001). The correlation coefficients were near perfect for both models whereas the TE was higher for 4C-DXA1 (2.88%) than 4C-DXA2 (2.60%). The SEE and 95% limits of agreement were lower for 4C-DXA2 (1.15% and ±2.5%, respectively) when compared to 4C-DXA1 (1.84% and ±3.8%, respectively). The proportional bias was statistically significant for 4C-DXA2 (p = 0.020), but not 4C-DXA1 (p = 0.183).

### Limits of agreement

**Fig 1:**
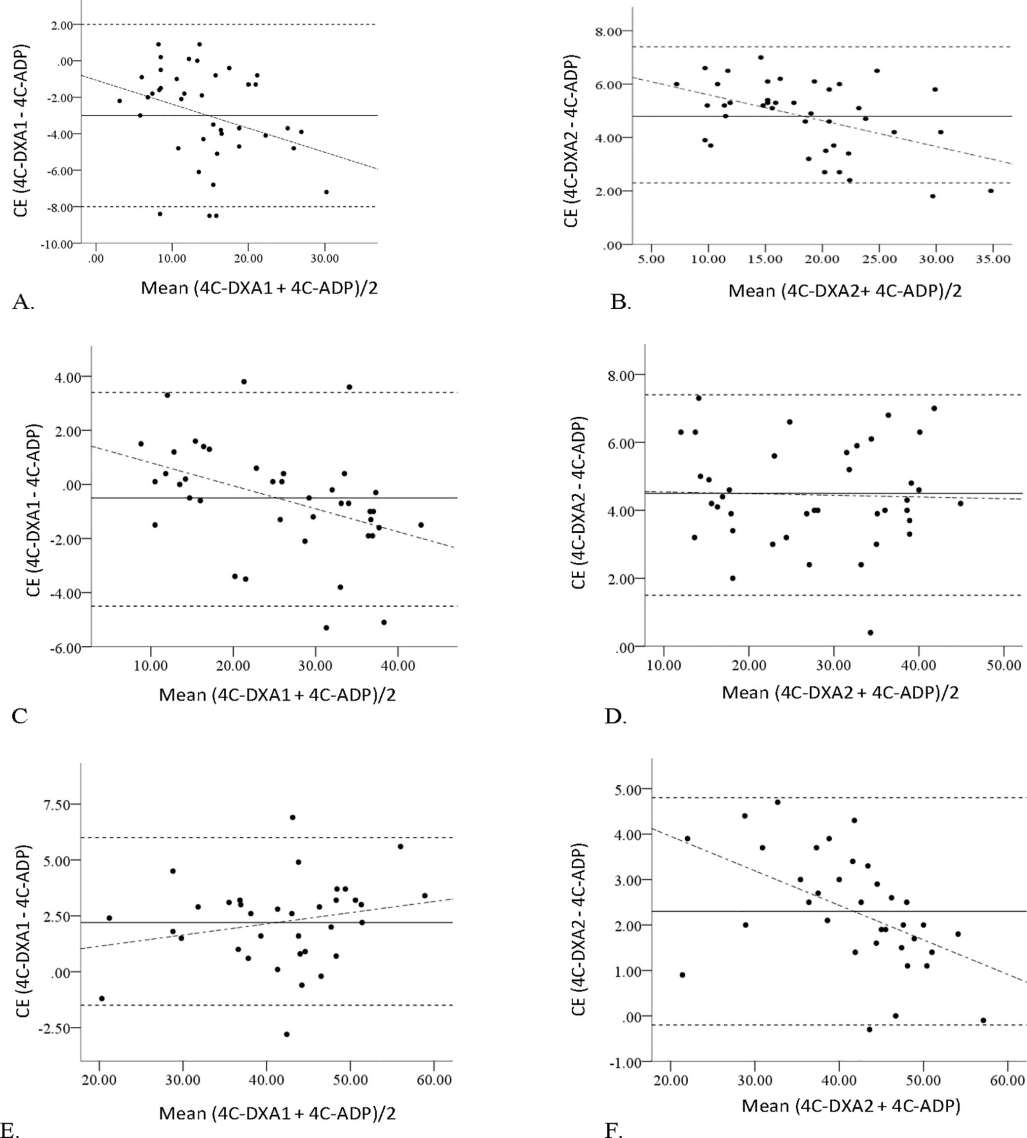
Bland-Altman plot for body fat percentage (BF%) in all three groups. The middle solid line represents the constant error between the 4C-DXA models and 4C-ADP BF% values. The 2 outside dashed lines indicate the 95% confidence interval of the bias (difference) and their means. The dashed-dotted line represents the linear regression fit line. Separate Bland-Altman plots depicting BF% in the 4C-DXA models are displayed for (A) 4C-DXA1 in NW; (B) 4C-DXA2 in NW_;_ (C) 4C-DXA1 in OW_BMI_; (D) 4C-DXA2 in OW_BMI_; (E) 4C-DXA1 in OW_BMI+WC_; (F) 4C-DXA2 in OWBMI+WC.

Finally, the Bland-Altman plot for BF% in all three groups is displayed in Fig 1.

## Discussion

The purpose of the current study was to determine the validity of the 4C model BF% estimates when using DXA-derived BV equations in adults with varying BMI and WC classifications. The systematic bias (i.e., CE) for 4C-DXA1 was rather large in NW and OW_BMI+WC_, but fairly small in OW_BMI_ as indicated by the trivial ES in the latter group. In contrast, the systematic bias was larger for 4C-DXA2 and implied that group BF% tended to be greatly overestimated in groups classified as NW and OW_BMI_ (CE = 4.9 and 4.5%, respectively). As a result, these findings highlight that practitioners should utilize caution when seeking to estimate group BF% estimates via DXA BV equations.

Although the group error for 4C-DXA1 was smaller than 4C-DXA2 for most comparisons, the latter method had lower individual error as indicated by the SEE and 95% limits of agreement. The larger individual error for 4C-DXA1 might be considered unacceptable when accuracy is highly desired. For example, the 95% limits of agreement revealed that 4C-DXA1 could underestimate BF% as much as 8.0% in participants classified as NW. Both equations yielded proportional bias in participants classified as NW. Furthermore, 4C-DXA1 and 4C-DXA2 revealed proportional bias in OW_BMI_ and OW_BMI+WC_, respectively. The negative regression coefficients (i.e., proportional bias) for all comparisons, except 4C-for OW_BMI_ via 4C-DXA2 and OW_BMI+WC_ via 4C-DXA _SMITH-RYAN_, suggests that the prediction models underestimated BF% relative to that derived using the 4C-ADP to a greater extent as BF% increased. Collectively, the present study findings demonstrate that both equations produced proportional bias in two of the three groups. Also systematic bias (i.e., CE) was more prevalent in NW and OW_BMI_ for 4C-DXA2 whereas larger individual error was present for 4C-DXA1. Thus, the use of DXA BV equations in a criterion 4C model is not recommended. Interestingly, 4C-DXA1 revealed the best validity in OW_BMI+WC_. For example, the systematic bias was trivial for 4C-DXA1 (CE = 2.2%). Furthermore, the individual error in OW_BMI+WC_ was narrow for 4C-DXA1 (TE = 2.88% SEE = 1.84%; 95% limits of agreement = ±3.8) and no proportional bias was present. This is a significant finding that demonstrates the ability of a clinical population (i.e. OW_BMI+WC_) to have a precise measurement of BF% with a simpler and more practical 4C model (i.e., 4C-DXA1).

The current study uniquely adds to previous research by stratifying subjects based upon BMI and WC while identifying how classification of these metrics affect the estimates of BF% when using DXA for BV in a 4C model. Since the derivation of DXA BV equations, limited research has been conducted to determine its validity when incorporated into a 4C model [9, 11–13]. Nickerson et al. [9] reported slightly larger SEEs (4.2%) and 95% limits of agreement (±8.2%) for 4C-DXA1 in a group of physically active men and women. It was reported that the individual error for the physically active men and women via 4C-DXA1 added minimal benefit over simpler two-compartment models [9]. Therefore, Nickerson et al. [9] concluded that 4C-DXA1 should not be used when high individual accuracy is desired. The current study supports this recommendation when using 4C-DXA1 in adults classified as NW. The 95% limits of agreement for 4C-DXA1 in the present study corresponded to 50% of the mean reference 4C-ADP BF% values and indicated that BF% could be underestimated as much as 8.0% in this population. Lastly, the 95% limits of agreement for 4C-DXA1 in the NW group (i.e., ±5.0%) is similar to what has been observed when comparing DXA in isolation (±5.9%) to a multi-compartment model [10]. Thus, 4C-DXA1 marginally increased the precision of simpler two-compartment model DXA measurements.

Recent findings from Blue et al. [13] revealed that 4C-DXA1 is valid in an OW_BMI_ population. However, the validity of 4C-DXA2 was not analyzed. The criteria for being classified as overweight was based solely on BMI whereas no information was provided on subjects’ WC [13]. The current study sought to advance these findings and determine whether overweight classification based solely on BMI yields different validity statistics than overweight classifications based on both BMI and WC. A limitation of BMI is that it is unable to distinguish between fat and lean mass. Physically active individuals can easily be misclassified as overweight via BMI due to large amount of lean mass and despite having recommended WC values and adiposity levels. For this reason BMI can often yield large error when used to estimate BF% [4]. Therefore, categorizing overweight individuals on the criteria of both BMI and WC prior to modified 4C model examinations (i.e., DXA-derived BMC and BV; BIA-derived TBW) might be more appropriate.

Reasons for systematic and proportional bias could be attributed to a number of factors. Both BV equations were derived using Hologic Discovery fan-beam DXA scanners [11, 12]. However, Wilson et al. [12] utilized the recommended National Health Examination Survey correction factor for the derivation of their equation and Smith-Ryan et al. [11] did not. The reasons for implementing the correction factor have previously been explained [20] and are one potential reason for the current study findings. Both study samples of Wilson et al. [12] and Smith-Ryan et al. [11] are relatively small (n = 25 and 100, respectively) compared to previous research studies that have derived body composition equations [21–23]. A limitation of the current study is sample size, thus future studies utilizing a larger and more diverse study sample might be needed for improved accuracy. Additionally, TBW was assessed with multi-frequency BIA and future studies should include an isotope dilution method. Finally, linear regression using the enter method was utilized to derive the DXA BV equations. Schumacker and Lomax [24] have previously expressed limitations of the enter method and recommend the use of an all-possible subset regression procedure in order to select the best set of independent predictor variables that have the highest R^2^ value. Thus, it is possible that an all-possible subset regression would have yielded different predictor coefficients, which might have reduced prediction error. This postulation is supported by the notion that BMC did not significantly add to the model when Wilson et al. [12] and Smith-Ryan et al. [11] derived their DXA BV equations (both p > 0.05). Future research should be conducted to further explore this notion and determine whether more accurate equations can be derived via this statistical procedure.

## Conclusions

In conclusion, the current study sought to determine the validity of 4C model BF% estimates when using DXA-derived BV equations in adults with varying BMI and WC classifications. Due to large CE, TE and proportional bias, 4C-DXA2 is not recommended for use in any group. Similarly, 4C-DXA1 should not be used in subjects classified as NW and OW_BMI_ due to large CE and 95% limits of agreement as well as proportional bias. In contrast, 4C-DXA1 had a small CE, TE, SEE and 95% limits of agreement and revealed no proportional bias in OW_BMI+WC_. For these reasons, 4C-DXA1 appears to be a suitable alternative in a modified 4C model for OW_BMI+WC_. As a result, practitioners are encouraged to use criteria for both BMI and WC when utilizing 4C-DXA1 in an overweight population. Future research is needed to derive a DXA BV equation that can be used in individuals with varying BMI and WC values.
